# Intraspecific Variation in Drought Response of Three Populations of *Cryptocarya alba* and *Persea lingue*, Two Native Species From Mediterranean Central Chile

**DOI:** 10.3389/fpls.2020.01042

**Published:** 2020-07-16

**Authors:** Carolina Alvarez-Maldini, Manuel Acevedo, R. Kasten Dumroese, Marta González, Eduardo Cartes

**Affiliations:** ^1^ Institute of Agri-food, Animal and Environmental Sciences (ICA3), Universidad de O Higgins, San Fernando, Chile; ^2^ Centro Tecnológico de la Planta Forestal, Instituto Forestal, San Pedro de la Paz, Chile; ^3^ Rocky Mountain Research Station, US Department of Agriculture, Forest Service, Moscow, ID, United States

**Keywords:** water deficit, relative growth rate, water use efficiency, gas exchange, population differences, restoration

## Abstract

An increase in the severity of drought events on Mediterranean climates highlights the need of using plant material adapted to drought during restoration efforts. Thus, we investigated between-population morpho-physiological differences in *Cryptocarya alba* and *Persea lingue*, two native species from Mediterranean central Chile, for traits that could effectively discriminate population performance in response to water restriction (WR) testing. Three populations from each species were subjected to WR treatment and physiological, morphological, and growth parameters were assessed at the beginning and at the end of the experiment. In *C. alba*, the most xeric population displayed smaller plants with mesophyllous leaves and lower photosynthetic rates indicating a resource saving strategy. Moreover, the xeric population performed better during WR than the most mesic populations, exhibiting higher water use efficiency (iWUE) and maintenance of growth rates. All *C. alba* populations responded equally to WR in terms of morphology and biomass partitioning. In contrast, differences among *P. lingue* populations were subtle at the morpho-physiological level with no apparent relation to provenance environmental conditions, and no morphological traits were affected by WR. However, in response to WR application, the most mesic population was, as observed through reduction in relative growth rates, more affected than xeric populations. We attribute such discrete differences between *P. lingue* provenances to the lower distributional range of selected populations. Our results show that relative growth rates in both species, and iWUE only in *C. alba*, exhibited population specific responses upon WR imposition; these results correspond with the environmental conditions found at the origin of each populations. Both traits could further assist in the selection of populations for restoration according to their response to water stress.

## Introduction

The world´s forests will need to cope and adapt to changes that are threatening their natural habitats (Allen et al., 2010; Gilliam, 2016). One significant disturbance expected by climate change is an increase in duration and frequency of drought events (Sheffield and Wood, 2008). Most models predict that water will become an even more limiting resource in Mediterranean-type sites under the increasing aridity (Sheffield and Wood, 2008). This is particularly serious for Mediterranean-type climates, already characterized by a dry, 1–5 month summer period with high temperatures imposing a physiological drought (Valladares et al., 2004).

Native ecosystems and their species distributions within the Chilean Mediterranean climate are not exempt from the effects of climate change related to increased drought (Garreaud et al., 2017). This has led to the development of restoration programs in ecosystems with high ecologic value (Bannister et al., 2018). Because knowledge concerning patterns of genetic variability in Chilean native species is scarce, the use of local plant material for restoration has been the customary approach. Concerns about the use of this approach are, however, increasing because of observations of lethal mutations among seedlings of native species, such as in *Cryptocarya alba* and *Persea lingue*, collected from local, but isolated populations (Alvarez et al., 2017). Plants from isolated populations are highly vulnerable to environmental changes because they are characterized by small population sizes, higher levels of inbreeding, and lower individual fitness and genetic variability (Aizen et al., 2002; Angeloni et al., 2011). It is expected that such plant material would have poor performance during drought events and have a lower capacity to adapt to changing climate (Valladares et al., 2007). The use of local plant material for restoration is common (Vander et al., 2010). Moreover, the selection of appropriate propagation stock based on their response to drought is advised, with the objective of replacing sensitive populations with ones that could respond better to forecasted climate change and reduce maladaptation risk (Bolte et al., 2009; Aitken and Whitlock, 2013). This intentional movement of populations to areas were future projected climate matches their current adapted environment (i.e., assisted migration) may be especially important for long-lived species threatened by fragmentation (Dumroese et al., 2015a). Until recently, it was believed that tree species from different populations responded equally to drought (Kawecki, 2008; Banta et al., 2012). However, habitat heterogeneity across species distribution can lead to variability in plant functional traits in response to environmental factors (Linhart and Grant, 1996; Hufford and Mazer, 2003), indicating that populations within a species experiencing different environmental conditions can differ in phenotypic characteristics and genetic structure (Linhart and Grant, 1996). This intraspecific variation in morpho-physiological traits in response to drought has been reported in a large number of tree species from Mediterranean climates (Gratani et al., 2003; Ramírez-Valiente et al., 2010; Kaluthota et al., 2015; Kerr et al., 2015; Ramírez-Valiente and Cavender-Bares, 2017).

Thus, higher degrees of intraspecific variation have been observed in individuals from contrasting environmental conditions. For example, rapid stomatal closure, an increase in water use efficiency (WUE), and a decrease in growth rate are commonly reported in individuals from drier climates (Kerr et al., 2015; Viger et al., 2016; Nguyen et al., 2017), although an opposite relationship between WUE and growth has been reported as well (Fardusi et al., 2016; Peguero-Pina et al., 2016). Such variation in response to drought is strongly associated with water availability in the population of origin and has shown to drive tree population differentiation (Zhang et al., 2004; Marchín et al., 2008; Aletá et al., 2009). This indicates high degrees of local adaptation, where individuals tend to display more relative fitness in their native environments (Savolainen et al., 2007). Local adaptation arises in response to differential selection pressures submitted through variable environmental conditions, especially in widely distributed tree species (Kawecki and Ebert, 2004; Savolainen et al., 2007), and constitutes a strategy to cope with environmental variability.

Hence, research regarding differential adaptive strategies within species and among populations increases the possibilities for selection of appropriate traits suitable for drier climates in future conditions; this is critical for afforestation and conservation purposes (Aitken et al., 2008; Grady et al., 2013). Adaptive responses to drought may be especially important for seedlings because of their susceptibility to water scarcity (Vennetier et al., 2007; Lloret et al., 2009) and because their survival is essential for the success of many restoration programs.

When patterns of genetic variability of species under restoration are unknown, the use of local plant material is advised (Vander et al., 2010). This is no exception for Chile, where the regulation for restoration efforts demands seeds or propagules from the nearest population of the sites to be restored. This approach does not consider plant material adaptation to the local environment, especially under increasing drought conditions of Mediterranean climates. Recent research in Chile suggests that fragmentation of native forests is leading to a possible decrease in genotypic variability (Echeverría et al., 2006), exemplified by an increase in albino-mutant phenotypes in seedlings of *P. lingue* and *C. alba* (Alvarez et al., 2017), increasing the need for research of intraspecific variation of these species under drought condition. Thus, an experiment under common garden conditions was established with three populations of *C. alba* and *P. lingue*. We evaluated physiological, morphological, and growth responses during a cycle of water restriction in both species from each population. We hypothesized that i) populations would display inter-specific differences at both the morphological and physiological levels, with higher differences in the most xeric populations, and ii) that population-specific differences in response to drought treatments would be apparent, especially in physiological parameters, with xeric populations displaying lower water use efficiency due to higher stomatal closure. To test our hypotheses, our objectives were i) to assess inter-population differentiation of morpho-physiological traits of both species and the effect of climate in such differences and ii) to test for morphological and physiological traits that could effectively differentiate population responses to water restriction.

## Materials and Methods

### Study Species, Sampled Populations, and Growing Conditions

The Chilean endemics *C. alba* and *P. lingue* are sclerophyllous shade tolerant species in the Laureaceae family. They share a large geographic range. *Cryptocarya alba* occurs from Coquimbo (lat 30°S) southward to the La Araucanía region (lat 38°S), while *P. lingue* is distributed from Valparaíso (lat 33°S) to the Los Lagos region (lat 40°S). Both species are highly abundant in the southernmost areas of their distribution (Ezcurra, 2010), usually growing on south-facing slopes or in humid ravines in the Mediterranean climate of central Chile (Niemeyer et al., 2002). We collected seeds from four locations in central Chile (Santiago, Nacimiento, Cayumanque, Santa Juana) that spanned an environmental gradient of xeric to mesic (**Table 1**). Within this gradient and for each species, we selected three populations. The most xeric collections for *C. alba* and *P. lingue* were Santiago and Nacimiento, respectively, whereas the most mesic collections were Cayumanque and Santa Juan, respectively (**Table 1**). The Cayumanque source for both species is especially important because of current restoration efforts; these sites have high levels of endemism and biodiversity but are threatened with severe fragmentation (Muñoz et al., 1996; CONAMA, 2002). We collected plant material from at least 30 mature individuals in all populations. Collections from sites other than Cayumanque were from native forests having a minimum area of 100 hectares and isolation from other sampling areas, with a minimum distance of 20 km between populations.

**Table 1:** Characteristics of the sites where *Cryptocarya alba* and *Persea lingue* populations were collected.

	Population			
	Santa Juana	Cayumanque	Nacimiento	Santiago
*Cryptocarya alba*	No	Yes	Yes	Yes
*Persea lingue*	Yes	Yes	Yes	No
Latitude	-37.317847	-36.694929	-37.480583	-33.483321
Longitude	-72.991540	-72.530487	-73.003362	-70.616602
Altitude (m.a.s.l)	333	771	645	716
P_mean_ (mm)	991	593	411	196
T_max_ (°C)	27.2	38.1	32.4	35.1
T_min_ (°C)	0.8	-2.2	-3.9	-5.3
ET (mm year^-1^)	941	1054	1102	1280

P_mean_, mean annual precipitation; T_max_, maximum annual temperature; T_min_, minimum annual temperature; ET, accumulated evapotranspiration (Penman-Monteith).

We collected seeds from February until May of 2016. After collection, seeds were cleaned, placed into plastic bags identified by species and population, and stored at 4°C until sowing. In July of 2016, seeds were sown into an outdoor growing facility at the Centro Tecnológico de la Planta Forestal, Instituto Forestal (lat -36.84°, long -73.13°), Biobío region, Chile. No pre-germination treatment was applied to *C. alba* seeds, while the exocarps and mesocarps of *P. lingue* seeds were removed. Seeds were sowed directly into expanded polystyrene trays (84 cavities per tray, each cavity 14 cm deep, and 130 ml) filled with composted *Pinus radiata* bark medium having total, aeration, and water retention porosity of 49%, 25%, and 24% respectively. Irrigation need was monitored with Decagon (Pullman, WA, USA) soil moisture sensors (ECH_2_O EC-5) and data logger (EM-50); sensor values for volumetric water content (m^3^m^-3^) were calibrated with gravimetric mass to estimate the percentage of available water (Dumroese et al., 2015b). During the germination phase until September of 2016, irrigation was applied daily with sprinklers. During the growth phase from October 2016 until December of 2017, irrigation was applied when the substrate lost 50% of the available water and fertigation events (fertilization mixed with water) were alternated with irrigation until the end of the growing phase. During the winter months (from April 2017 until September 2017) no fertilization was added and rainfall provided sufficient irrigation. Fertilization consisted of constant rates of nitrogen, phosphorous, potassium, calcium, magnesium, and sulfur at 300, 150, 180, 100, 80, and 115 mg L^-1^, respectively, using calcium nitrate (CaNO_3_), magnesium sulfate (MgSO_4_), ammonium nitrate (NH_4_NO_3_), potassium phosphate monobasic (KH_2_PO_4_), and urea [CO(NH_2_)_2_]. Micronutrients fertilization consisted of constant rates of iron, manganese, copper, and zinc at 6, 4, 0.5, and 6 mg L^-1^, respectively, using iron sulfate (FeSO_4_), manganese sulfate (MnSO_4_), copper sulfate (CuSO_4_), and zinc sulfate (ZnSO_4_).

### Water Restriction Experiments

**Figure 1:**
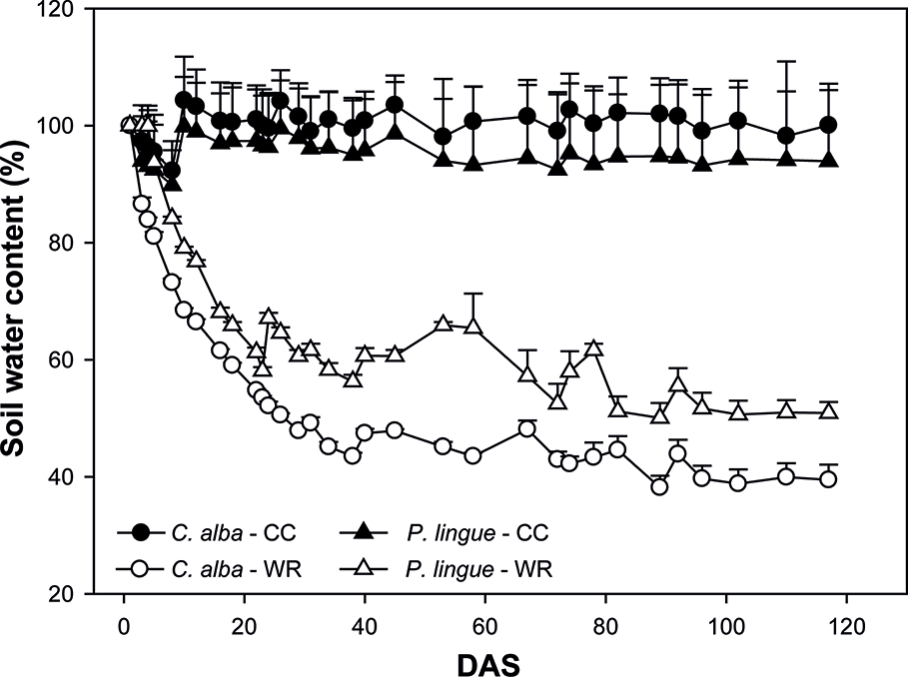
Evolution of soil water content (%) during the water restriction experiment for *Cryptocarya alba* (circles) and *Persea lingue* (triangles) plants at container capacity (CC, black symbols) and in the water restriction (WR, white symbols) treatment. DAS, days after onset of stress treatment. Symbols represent mean + SE.

In January 2018, 50 plants from each population and species were transferred to plastic pots of 10 L capacity (29 cm upper diameter and 22 cm height). Each pot was filled with an equal amount of soil from Cayumanque as substrate. The soil is of metamorphic origin and presents an average pH of 6.1 and an organic matter content of 7.9%. Levels of nitrogen and phosphorous are 2.1 and 33.7 mg kg^-1^, respectively. The experiment was done inside a greenhouse (42 m^2^) under semi-controlled conditions. Treatments and populations were assigned randomly within the greenhouse. During the course of the experiment, the minimum and maximum temperature and relative humidity were 7.3–27.8°C and 49%–99%, respectively. Before onset of the water restriction experiments, plants were acclimated to their pots for one month. During this time, pots were irrigated every 2 days to container capacity and we monitored volumetric soil water content (VSWC) (m^3^m^-3^) every 30 min as described above (see evolution of volumetric water content at **Figure S1**, in **Supplementary Material**). Additionally, VSWC (%) was estimated by comparing the weights of pots at container capacity (pot_wet_) with pots with substrate dried in an oven until constant weight (pot_dry_) using this equation: [(pot-pot_dry_)/(pot_wet_-pot_dry_)] *x* 100 (Peuke et al., 2002). After the month of acclimation, plants of each species were divided into two water availability treatments: control and restricted. Control plants (CC) were irrigated regularly to maintain the water content of the substrate at container capacity during the entire experiment. Plants in the water restriction treatment (WR) had irrigation withheld until day 40, at which point VSWC reached 43.0 ± 0.8% and 53.7 ± 1.2% in *C. alba* and *P. lingue*, respectively. This VSWC was maintained by weighing the pots every two days and adding water to the substrate to reach desired levels until day 120 (**Figure 1**).

### Physiological, Morphological, and Growth Measurements

One month after transplanting, we made an initial measurement of the seedlings. We randomly sampled six seedlings (each seedling was a replicate) from each species (2) and population (3) combination (36 seedlings total) for physiological and morphological traits.

For physiological traits, we measured gas exchange, chlorophyll fluorescence, mid-day water potential, and relative water content. We randomly selected fully developed leaves from the upper third of the stem (i.e., leaves present before onset of the WR treatment). Gas exchange was measured on one leaf per seedling with a LI-6400XT portable photosynthesis system (LI-COR Instruments, Lincoln, NE, USA) having a CO_2_ concentration inside the cuvette set to 400 µmol mol^-1^, leaf temperature maintained at 25 ± 1°C, and leaf illumination of 1500 µmol m^-2^s^-1^ of photosynthetic photon flux density (PPFD) provided using a mixed red/blue light emitting diode installed on top of the cuvette; measurement duration was 10 to 15 min. Net photosynthesis based on leaf area (A_N_), stomatal conductance (gs), intercellular concentration of CO_2_ (Ci), and transpiration rate (E) were obtained. Intrinsic water use efficiency (iWUE) was calculated as the ratio between A_N_ and gs.

We measured chlorophyll fluorescence on one leaf using a pulse-amplitude modulated fluorimeter (FMS2, Hansatech Instruments, Norfolk, England). Leaves were covered with a clamp and adapted to dark for 30 min before fluorescence measurements. The protocol for chlorophyll fluorescence curves was performed according to Alvarez et al. (2012) with slight modifications. The saturating pulses corresponded to the following PPFDs: 14, 35, 86, 167, 562, 888, 1287, and 1824 µmol m^-2^s^-1^. Maximum efficiency of photosystem II (F_v_/F_m_) and electron transport rate (ETR) were calculated according to equations 1 and 2, respectively:

(1) $$\frac{F_{v}}{F_{m}}=\frac{\left(F_{m}-F_{o}\right)}{F_{m}}$$

(2) ETR=PFD×0.5×ΦPSII×0.84

where ΦPSII is the effective quantum yield of PSII, the 0.5 factor assumes that the efficiency between PSI and PSII is equal and that the incident light is equally distributed between them, and the 0.84 factor is the mean value of absorbance for green leaves. The non-photochemical quenching (NPQ) and the photochemical quenching (qP) were calculated according to equations 3 and 4, respectively:

(3) $$\mathrm{NPQ}=\frac{\left(F_{m}-F_{m^{\prime }}\right)}{F_{m^{\prime }}}$$

(4) $$\mathrm{qP}=\frac{\left(F_{m^{\prime }}-F_{s}\right)}{F_{m^{\prime }}-F_{o^{\prime }}}$$

After completing the gas exchange and chlorophyll fluorescence measurements, we randomly selected two fully expanded leaves from the upper third of each seedling for mid-day water potential (Ψ_min_) and two more for relative water content (RWC) assessment (Nguyen et al., 2017). For mid-day water potential, leaves were excised with a razor blade at petiole level between 11:00 PM and 14:00 PM and Ψ_min_ was immediately measured with a Scholander pressure chamber (PMS Instruments, Albany, OR, USA) (Scholander et al., 1965). Harvested leaves were retained to be included in the morphological assessments.

For morphological measurements, plants were removed from pots and separated into leaves, roots, and stems. Roots were gently washed to remove soil. Individual leaves (including those from the physiological tests) were photographed and analyzed using an image analysis software (ImageJ, Rasband/NIH, Bethesda, MD, USA) to obtain total leaf area. Individual leaf area was calculated by dividing total leaf area by the number of leaves of each sample. Stomatal density was determined using the impression approach. The abaxial size of each leaf was cleaned and carefully smeared with fingernail varnish in the area between the leaf margin and the mid-vein. A thin film was peeled off, placed on a glass slide, and covered with a cover slip. The slides were mounted on a microscope (Olympus CX31, Tokyo, Japan) and photographed with Micrometrics SE software v2.7 (Accu-Scope, Commack, NY, USA). Photos were analyzed with the ImageJ software (Rasband/NIH, Berthesda, MD, USA); an area of 100 µm^2^ was marked and the total number of stomas inside the area were counted. Each plant component was oven dried at 100°C to constant weight and then root, leaf, and stem dry mass were obtained. Total plant dry mass was calculated as the sum of all plant parts. Specific leaf area (SLA) was calculated as the ratio between leaf area and leaf dry mass, while leaf area ratio (LAR) was calculated as the ratio between leaf area and total dry mass. Also, leaf, root, and stem mass ratio were calculated by dividing the mass of each plant component by total dry mass. Wood density was calculated as oven-dry mass of a 2-cm long segment of the basal part of each plant divided by its green volume (Chave et al., 2009).

At the end of the experiment (120 days after onset of the WR treatment), we randomly selected 6 seedlings from each species (2) x irrigation treatment (2) x population (3) combination (72 plants total) for the final measurement. The same suite of physiological and morphological measurements were completed as described above. For the physiological and stomatal density measurements, we sampled fully expanded leaves that were initiated during the WR treatment.

Changes in morphological traits between the initial (pre-WR treatment) and final (post-WR treatment) measurements allowed us to assess growth. Relative growth rate (RGR) and net assimilation rate (NAR) were calculated with total dry mass (TM) from the initial and final measurement times according to equations 5 and 6 (Galmés et al., 2005):

(5) $$RGR=\frac{log_{e}TM_{2}-log_{e}TM_{1}}{t_{2}-t_{1}}$$

(6) $$NAR=\frac{\left(TM_{2}-TM_{1})\times (log_{e}LA_{2}-log_{e}LA_{1}\right)}{\left(t_{2}-t_{1})\times (LA_{2}-LA_{1}\right)}$$

where t_1_ and t_2_ correspond to the initial and final measurement times, respectively.

### Data Analysis

Most of the physiological and all of the morphological variables were analyzed by species with a repeated measures model (Kuehl, 2001), modeling the variance and covariance structure with a 95% level of confidence. Statistical differences between means were performed with a Tukey (HSD) test for multiple comparisons, using the PROC MIXED procedure (SAS Institute Inc., Cary, NC, USA). The effect of the different factors was assessed using the following general linear model (GLM):

(7) $$y_{ijkl}=\mu +\alpha_{i}+\beta_{j}+\gamma_{k}+{\left(\alpha \beta \right)}_{ij}+{\left(\alpha \gamma \right)}_{ik}+{\left(\beta \gamma \right)}_{jk}+{\left(\alpha \beta \gamma \right)}_{ijk}+e_{ijkl}$$

where γ*_ijkl_* is the value of de dependent variable in plant *l* measure in time (*i*=1, 2), in the *j* population (*j*=1, 2, 3) in the *k* irrigation treatment (*k*=1, 2). µ is the overall mean; α, β, and γ are the fixed effects for time, population, and water restriction treatment respectively; the double and triple terms represent interactions; and *e* is the error term for the hypothesis *e_ijkl_* $∼N(0,\sigma_{e}^{2})$.

Relative growth rate and net assimilation rate were assessed with two-way analysis of variance (ANOVA) through a PROC GLM procedure (SAS Institute). Differences among means were determined with a Tukey (HSD) test for multiple comparisons.

Chlorophyll fluorescence curves to different PPFD were modeled using the PROC NLIN procedure (SAS Institute Inc.) using the Gauss-Newton method through a derivative-free algorithm. The variables ETR, NPQ, and qP in response to different PPFD were modeled using the Michaelis-Menten model:

(8) $$y=(\beta_{1}\times PPFD)/(\beta_{2}+PPFD)$$

The variables ΦPSII and qP were modeled using the first-order decay kinetics model:

(9) $$y=\beta_{1}\times EXP(-\beta_{2}\times PPFD)+\beta_{3}$$

The effect of WR was evaluated by population and species to models level using the extra sums of squares principle (Bergerud, 1996). Visualizations were made using SigmaPlot 10 (Systat Software Inc., San Jose, CA, USA).

## Results

### Gas Exchange and Chlorophyll Fluorescence

**Figure 2:**
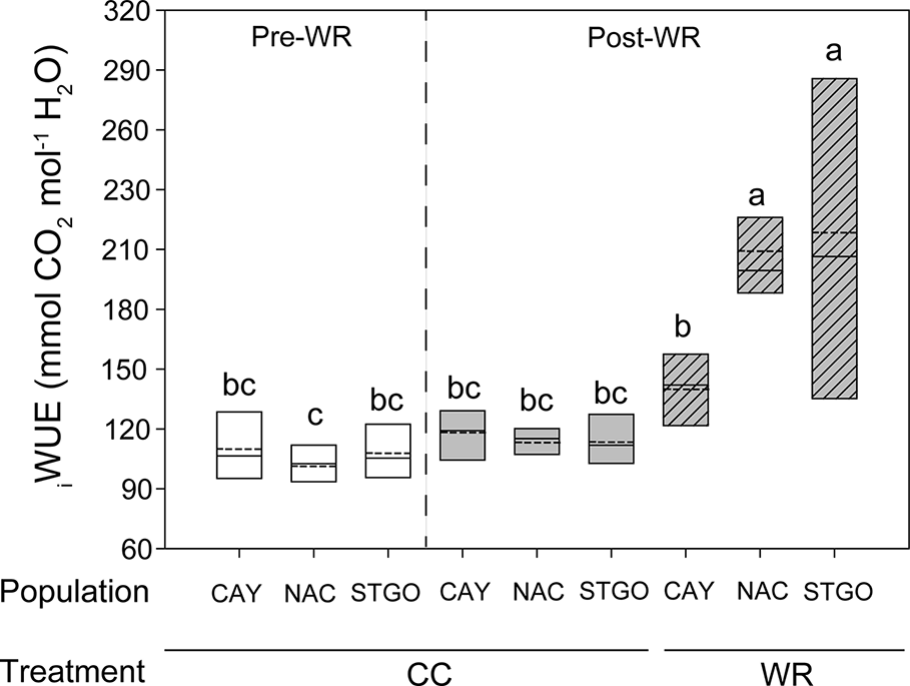
Significant triple interaction between measurement time, population, and water restriction treatment affected intrinsic water use efficiency (iWUE, mmol CO_2_ mol^-1^ H_2_O) in *Cryptocarya alba* plants. Bottom and top boundaries of the boxes represent the 25^th^ and 75^th^ percentiles, respectively. The solid line in the center of each box represents the mean value and the dashed line represents the median value. Different letters indicate significant differences among measurement time, populations, and water restriction treatment according to Tukey at p<0.05. Pre-WR, measurement before the onset of water restriction; Post-WR, measurement at the end of the experiment; CC, container capacity; WR, water restriction; CAY, Cayumanque; NAC, Nacimiento; STGO, Santiago.

In *C. alba*, population and the WR treatment independently affected A_N_, gs, and E (**Table 2**). Before the application of the WR treatment, Santiago presented significantly lower A_N_ than Cayumanque and Nacimiento (2.05 ± 0.19 vs. 6.65 ± 0.58 vs. 6.45 ± 0.64 µmol CO_2_ m^-2^s^-1^, respectively). At the conclusion of the WR treatment, we noted this relationship changed due to a significant interaction between population *x* measurement time; Santiago increased A_N_ and no significant differences were observed with Cayumanque or Nacimiento (6.42 ± 0.78 vs. 6.16 ± 0.9 vs. 4.23 ± 0.8 µmol CO_2_ m^-2^s^-1^, respectively); gs and E followed the same pattern as A_N_. The application of WR significantly decreased A_N_ compared to CC plants (3.31 ± 0.36 vs. 7.90 ± 0.49 µmol CO_2_ m^-2^s^-1^) in all populations. Similarly, WR decreased gs and E compared to CC plants (0.19 ± 0.03 vs. 0.66 ± 0.05 mol m^-2^s^-1^ and 0.94 ± 0.07 vs. 0.23 ± 0.04 mmol H_2_O m^-2^s^-1^, respectively). We observed a significant triple interaction between factors that affected iWUE (**Table 2**). Descriptively, no differences were observed between CC plants from both measurement times and populations, but the WR treatment increased iWUE on plants from Nacimiento and Santiago whereas no change was observed for Cayumanque (**Figure 2**).

**Table 2:** Summary of three-way repeated measures ANOVA p-values for treatment effects on net photosynthesis (A_N_), stomatal conductance (gs), intercellular CO_2_ concentration (Ci), transpiration rate (E), and intrinsic water use efficiency (iWUE) in *Cryptocarya alba* and *Persea lingue* plants.

Species	Source of variation	A_N_	gs	Ci	E	iWUE
*Cryptocarya alba*	MT	0.1904	0.5265	**0.0178**	0.0863	**<0.0001**
	P	**0.0003**	**0.0019**	0.1114	**0.0012**	0.2235
	WR	**<0.0001**	**<0.0001**	0.2671	**<0.0001**	**<0.0001**
	P x MT	**<0.0001**	**<0.0001**	0.2839	**<0.0001**	**0.0351**
	MT x WR	**<0.0001**	**<0.0001**	0.2671	**<0.0001**	**<0.0001**
	P x WR	0.9112	0.9383	0.4182	0.9833	0.0918
	P x MT x WR	0.9112	0.9383	0.4182	0.9833	**0.0364**
*Persea lingue*	MT	**0.0001**	**0.0208**	**<0.0001**	0.0518	**<0.0001**
	P	0.8755	0.1514	0.1857	0.2831	0.8530
	WR	**<0.0001**	0.1537	0.4489	0.1146	0.6613
	P x MT	**<0.0001**	**0.0021**	**0.0001**	**0.0009**	0.1765
	MT x WR	**<0.0001**	0.1691	0.4489	0.1410	0.5740
	P x WR	**0.0308**	0.7302	0.7815	0.5438	0.9761
	P x MT x WR	**0.0308**	0.7471	0.7815	0.5893	0.9609

Significant p-values (p<0.05) are in bold. Each species was analyzed separately. MT, measurement time; P, population; WR, water restriction treatment.

**Figure 3:**
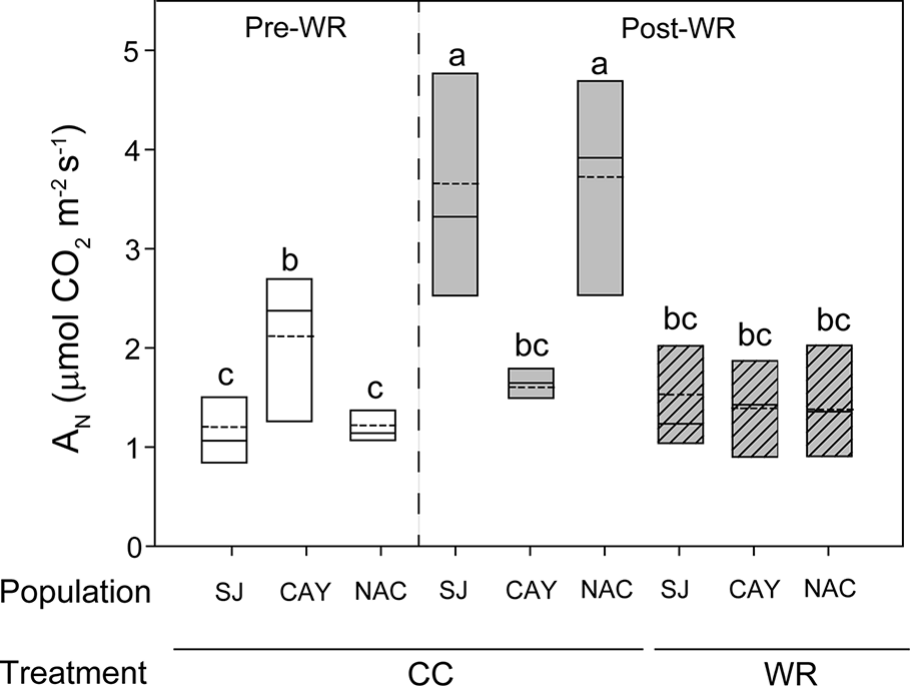
Significant triple interaction between measurement time, population, and water restriction treatment affected net photosynthesis (A_N_, µmol CO_2_ m^-2^s^-1^) in *Persea lingue* plants. Bottom and top boundaries of the boxes represent the 25^th^ and 75^th^ percentiles, respectively. The solid line in the center of each box represents the mean value and the dashed line represents the median value. Different letters indicate significant differences among measurement time, populations, and water restriction treatment according to Tukey at p<0.05. Pre-WR, measurement before the onset of water restriction; Post-WR, measurement at end of experiment; CC, container capacity; WR, water restriction; SJ, Santa Juana; CAY, Cayumanque; NAC. Nacimiento.

In *P. lingue*, we observed a significant interaction between measurement time *x* population (**Table 2**) for gs, Ci, and E (data not shown); WR treatment had no significant effect in any variable. Before the WR treatment, Cayumanque displayed higher A_N_ than Nacimiento and Santa Juana plants (2.12 ± 0.29 vs. 1.22 ± 0.1 vs. 1.20 ± 0.2 µmol CO_2_ m^-2^s^-1^, respectively); the same trend was also observed in gs (0.022 ± 0.004 vs. 0.012 ± 0.002 vs. 0.014 ± 0.001 mmol H_2_O m^-2^s^-1^, respectively). These parameters decreased during the WR treatment, but mainly affected Cayumanque plants. In contrast, a significant triple interaction among measurement time *x* population *x* WR treatment was found for A_N_ (**Table 2**). Net photosynthesis remained unaffected in Cayumanque plants as measured pre- and post-WR treatment, while it decreased in WR plants from Nacimiento and Santa Juana compared to CC plants (**Figure 3**)

**Figure 4:**
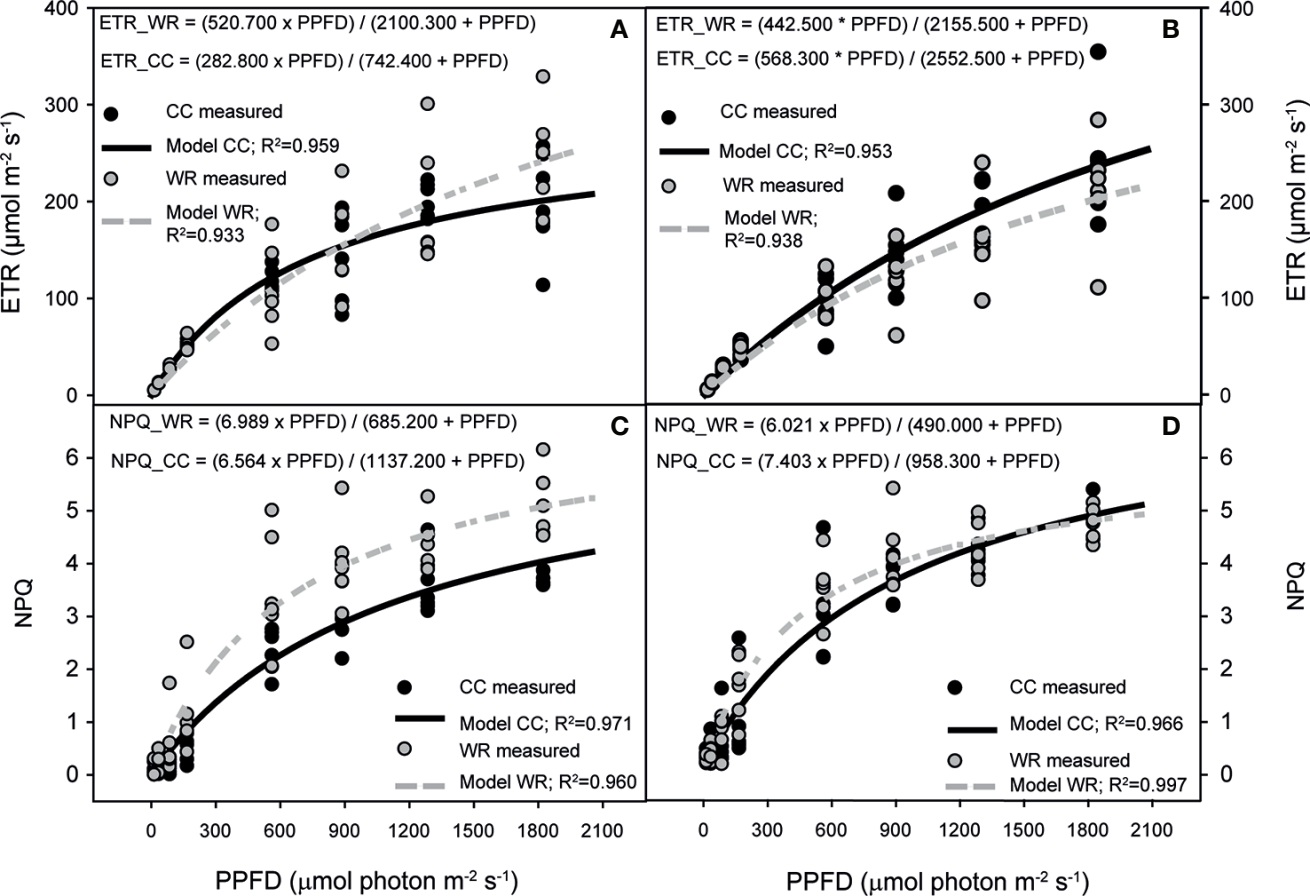
Effect of increasing photosynthetic photon flux density (PPFD, µmol photon m^-2^s^-1^) on electron transport rate (ETR, µmol m^-2^s^-1^) **(A**, **B)** and non-photochemical quenching (NPQ) **(C, D)** of *Cryptocarya alba* plants from Santiago **(A)**, Nacimiento **(B, D)** and Cayumanque **(C)** under the water restriction (WR) and container capacity (CC) treatments. Dots indicate measured data (CC in black, WR in gray) and lines indicate modelled data (CC in continuous black line, WR in dashed gray line). Adjusted model for CC and WR treatments for each variable and population are shown on each panel.

Regarding chlorophyll fluorescence curves, the effect of WR was analyzed separately by population and species. In *C. alba*, WR affected the responses of ETR in Santiago and Nacimiento but not in Cayumanque (p=0.027, p=0.019, and p=0.145, respectively). In Santiago, WR increased ETR compared to CC plants (**Figure 4A**), whereas the opposite was observed in Nacimiento plants (**Figure 4B**). Non-photochemical quenching was affected by WR treatment on Cayumanque (**Figure 4C**) and Nacimiento plants but not on plants from Santiago (p<0.0001, p=0.017, and p=0.157, respectively). Water restriction treatment increased NPQ in Cayumanque and Nacimiento compared to CC plants, although in Nacimiento plants NPQ increased transiently at PPFDs between 167 and 888 µmol photon m^-2^s^-1^ (**Figure 4D**). Also, ϕPSII decreased in Santiago plants subjected to WR at PPFD between 86 and 1287 µmol photon m^-2^s^-1^ compared to CC plants, while no effect was observed in Cayumanque or Nacimiento (p=0.036, p=0.256, and p=0.054, respectively). Photochemical quenching was not influenced by WR in any population.

**Figure 5:**
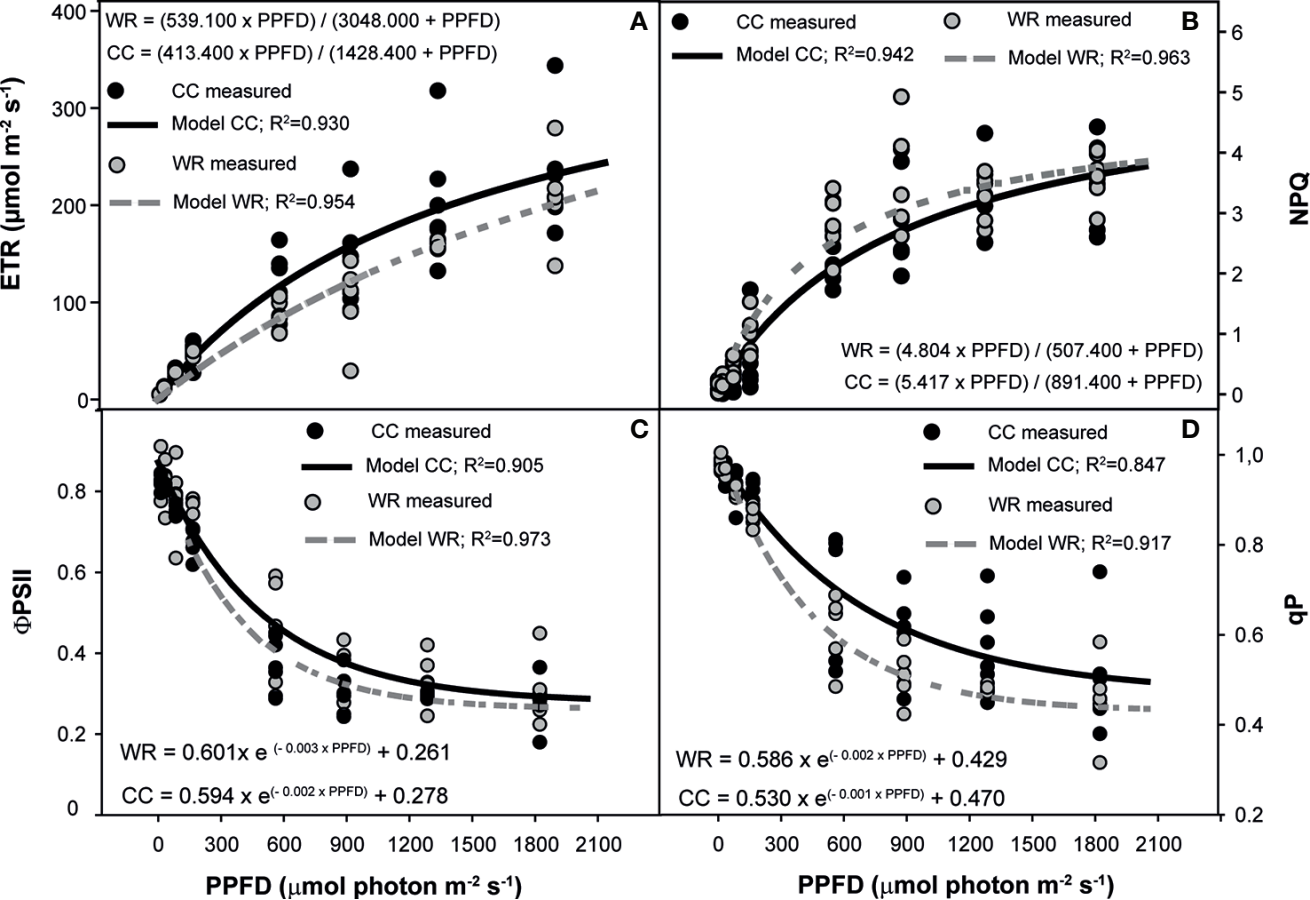
Effect of increasing photosynthetic photon flux density (PPFD, µmol photon m^-2^s^-1^) on electron transport rate (ETR, µmol m^-2^s^-1^) **(A)**, non-photochemical quenching (NPQ) **(B)**, photochemical efficiency of PSII (ϕPSII) **(C),** and photochemical quenching (qP) **(D)** of *Persea lingue* plants from Cayumanque under the water restriction (WR) and container capacity (CC) treatments. Dots indicate measured data (CC in black, WR in gray) and lines indicate modelled data (CC in continuous black line, WR in dashed gray line). Adjusted model for CC and WR treatments for each variable and population are shown on each panel.

In *P. lingue*, WR significantly affected the responses of ETR, NPQ, ϕPSII, and qP in plants from Cayumanque (p=0.001, p=0.031, p=0.045, and p=0.035, respectively). In Nacimiento only NPQ (p=0.035) was influenced by WR, while photochemistry was not altered by WR in plants from Santa Juana. In plants from Cayumanque, the application of WR decreased ETR, ϕPSII, and qP (**Figures 5A, C, D** **)** and increased NPQ (**Figure 5B**) compared to CC plants. In contrast, plants from Nacimiento the WR treatment decreased NPQ in comparison to CC plants.

### Plant Water Status

In *C. alba*, population and measurement time interacted to affect mid-day water potential (Ψ_min_; p=0.0094). Before onset of the WR treatment, Ψ_min_ was significantly lower in the Santiago population compared to the Cayumanque and Nacimiento populations (-0.78 ± 0.03 vs. -0.63 ± 0.03 vs. -0.55 ± 0.04 MPa, respectively). At the end of the experiment and independent of population, WR significantly (p<0.0001) decreased Ψ_min_ compared to CC plants (-0.43 ± 0.03 vs. -1.56 ± 0.05 MPa). In *P. lingue*, WR treatment and measurement time interacted to affect Ψ_min_ (p<0.0001). At the beginning of the experiment Ψ_min_ was -0.33 ± 0.03 MPa for all populations and remained relatively constant in CC plants throughout the experiment (-0.34 ± 0.04 MPa), whereas it decreased for WR plants to -1.59 ± 0.04 MPa.

For relative water content in *C. alba*, a small but significant (p=0.0006) variation was observed among populations: Cayumanque was lower than Nacimiento and Santiago (91.2 ± 0.4 vs. 92.6 ± 0.4 vs. 93.5 ± 0.5%, respectively). Similarly in *P. lingue*, relative water content was affected by population (p=0.003), with Nacimiento displaying lower values than Cayumanque and Santa Juana (88.1 ± 0.6 vs. 91.1 ± 0.6 vs. 91.0 ± 0.6%). The WR treatment had no effect on this parameter in either species (p=0.073 in *C. alba*, p=0.914 in *P. lingue*).

### Biomass Partitioning, Stomatal Density, and Relative Growth

**Figure 6:**
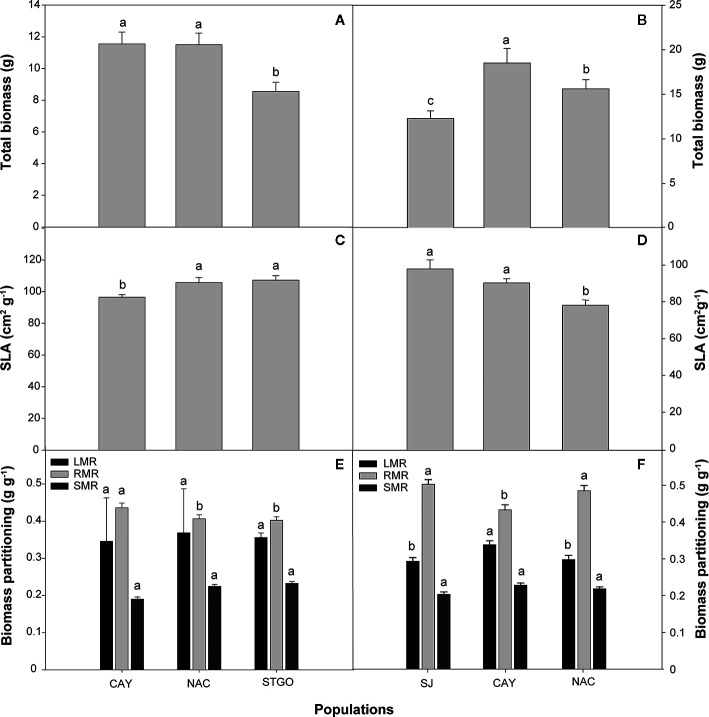
Effect of population on total biomass (g), specific leaf area (SLA, cm^2^g^-1^), and biomass partitioning (g g^-1^) of *Cryptocarya alba* **(A, C,** **E)** and *Persea lingue* **(B, D, F)** plants. Bars indicate means + SE. Different letters indicate significant differences according to Tukey at p<0.05. CAY, Cayumanque; NAC, Nacimiento; STGO, Santiago; SJ, Santa Juana; LMR, leaf mass ratio; RMR, root mass ratio; SMR, stem mass ratio.

In *C. alba*, population significantly affected all morphological parameters, including those associated with biomass partitioning, except total leaf area, leaf area ratio, and leaf mass ratio (**Table 3**; see **Supplemental Table 1** for mean morphological values). For these exceptions, we observed high standard deviations around the means (see **Supplemental Table 1**), which resulted in large p-values for the various interactions of population, treatment, and measurement time (**Table 3**). Although a Pearson’s correlation analysis revealed no significant correlation between individual leaf area and stomatal density (r=-0.11, p=0.528), we observed significant differences in stomatal density (p=0.0002). Plants under WR had higher stomatal density than CC plants (35.8 ± 1.3 vs. 29.8 ± 0.8 stomas 100 µm^-2^). Individual leaf area was only significantly affected by population (**Table 3**), higher values were observed in Nacimiento (7.31 ± 1.69 cm^2^ leaf^-1^) followed by Cayumanque (5.40 ± 1.46 cm^2^ leaf^-1^) and Santiago (3.77 ± 0.83 cm^2^ leaf^-1^) Total biomass was significantly higher in Cayumanque and Nacimiento compared to Santiago (**Figure 6A**), while specific leaf area was significantly higher in Nacimiento and Santiago than Cayumanque (**Figure 6C**). In relation to biomass partitioning, we observed no differences in leaf mass ratio and stem mass ratio between populations, but the ratio for root mass was higher in Cayumanque when compared to Nacimiento and Santiago (**Figure 6E**). Compared to CC plants, those under WR increased their root mass ratio (WR = 0.47 ± 0.01 vs. CC = 0.43 ± 0.01 g g^-1^) and decreased their leaf dry matter (WR = 3.96 ± 0.28 vs. CC = 5.05 ± 0.43 g). A significant interaction between population and WR treatment affected stem dry mass (**Table 3**); Nacimiento and Cayumanque populations had more dry mass in the CC treatment (4.7 ± 0.45 and 2.90 ± 0.30 g, respectively) than those in the WR treatment (3.28 ± 0.34 and 1.76 ± 0.15 g, respectively), but plants from Santiago were unaffected by water supply (WR = 2.75 ± 0.21 g and CC = 2.37 ± 0.22 g).

**Table 3:** Summary of three-way repeated measures ANOVA p-values for treatment effects on total biomass (TM), leaf dry mass (LDM), shoot dry mass (SDM), root dry mass (RDM), leaf area (LA), individual leaf area (ILA), specific leaf area (SLA), leaf area ratio (LAR), leaf mass per area (LMA), leaf mass ratio (LMR), shoot mass ratio (SMR), root mass ratio (RMR), relative growth rate (RGR), net assimilation rate (NAR), and wood density (WD) in *Cryptocarya alba* and *Persea lingue* plants.

Species	Source of variation	TM	LDM	SDM	RDM	LA	ILA	SLA	LAR	LMA	LMR	SMR	RMR	RGR	NAR	WD
*C. alba*	MT	**0.0001**	0.2118	**0.0001**	**0.0001**	0.8913	0.8433	**0.0006**	**0.0001**	**0.0002**	**0.001**	0.3774	**0.0001**	—	—	**0.0001**
	P	**0.0001**	**0.0001**	**0.0001**	**0.0001**	0.1718	**0.0001**	**0.007**	0.0856	**0.0133**	0.7854	**0.0001**	**0.0104**	**0.0001**	**0.0001**	**0.0390**
	WR	0.0557	**0.0130**	**0.0199**	0.4781	0.2357	0.1993	0.2257	0.0640	0.2414	0.0743	0.2230	**0.0188**	**0.0002**	**0.0005**	**0.0001**
	P x MT	**0.0001**	**0.0001**	**0.0074**	**0.0043**	0.2079	0.2617	0.2192	0.1201	0.2850	0.2374	0.2757	0.6230	**—**	**—**	0.6778
	MT x WR	0.0557	**0.0130**	**0.0199**	0.4781	0.2357	0.2313	0.2257	0.0640	0.2414	0.1831	0.1217	**0.0188**	—	—	**0.0001**
	P x WR	0.0608	0.0980	**0.0416**	0.1375	0.4346	0.1576	0.9324	0.6262	0.9979	0.3794	0.6557	0.7549	**0.0002**	**0.0005**	0.6091
	P x MT x WR	0.0608	0.0980	**0.0416**	0.1375	0.4346	0.1992	0.9324	0.6262	0.9979	0.5861	0.5040	0.7549	—	—	0.6091
*P. lingue*	MT	**0.0006**	0.2597	0.0792	**0.0001**	0.5675	0.1662	0.8333	**0.0046**	0.3752	**0.0001**	**0.0002**	**0.0001**	—	—	**0.0003**
	P	**0.0013**	**0.0003**	**0.0001**	**0.0336**	0.2223	**0.0121**	**0.0007**	**0.0017**	**0.0001**	**0.001**	**0.0014**	**0.0001**	**0.0001**	**0.0022**	**0.0026**
	WR	0.2528	0.7143	0.3559	0.086	0.9188	0.8349	0.4164	0.1371	0.7526	0.1270	0.6639	0.0988	**0.0001**	**0.0118**	0.7724
	P x MT	0.3383	0.7411	0.2825	0.1468	0.9637	0.1339	0.1097	0.1539	**0.034**	0.0786	0.4022	0.5485	—	—	0.7846
	MT x WR	0.2528	0.7143	0.3559	0.0860	0.9188	0.8349	0.4164	0.1371	0.7526	0.1270	0.7585	0.0988	—	—	0.783
	P x WR	0.7512	0.8795	0.5293	0.8010	0.9415	0.7518	0.8736	0.7299	0.7711	0.8513	0.4882	0.8622	**0.0001**	0.4646	0.8354
	P x MT x WR	0.7512	0.8795	0.5293	0.8010	0.9415	0.7518	0.8736	0.7299	0.7711	0.8513	0.6958	0.8622	—	—	0.8495

Significant p-values (p<0.05) are in bold. Each species were analyzed separately. MT, measurement time; P, population; WR, water restriction treatment.

**Figure 7:**
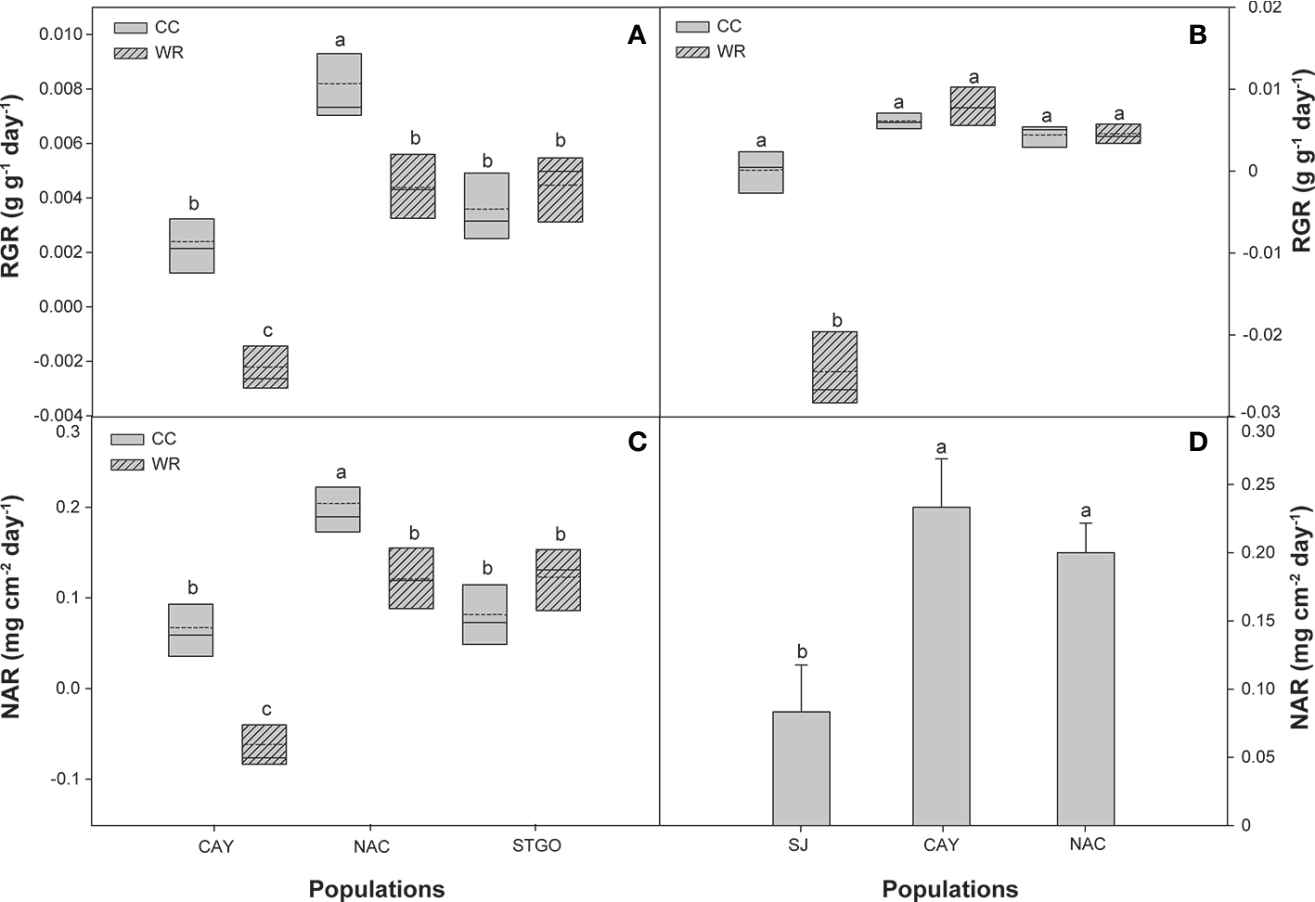
Effect of population and irrigation treatment on relative growth rate (RGR, g g^-1^ day^-1^) and net assimilation rate (NAR, mg cm^2^ day^-1^) of *Cryptocarya alba* **(A**, **C)** and *Persea lingue* **(B, D)** plants. In panels **(A–C)**, the bottom and top boundaries of the boxes represent the 25^th^ and 75^th^ percentiles, respectively. The solid line in the center of each box represents the mean value and the dashed line represents the median value. Different letters indicate significant differences among populations and irrigation treatments according to Tukey at p<0.05. In panel D, bars indicate means + SE. Different letters indicate significant differences according to Tukey at p < 0.05. CAY, Cayumanque; NAC, Nacimiento; STGO, Santiago; SJ, Santa Juana; WR, water restriction; CC, container capacity.

Wood density in *C. alba* was affected by population and level of water restriction (**Table 3**), with WR plants having a greater density than CC plants (0.60 ± 0.01 vs. 0.49 ± 0.01 g cm^-3^, respectively) and Cayumanque having greater wood density than Nacimiento and Santiago (0.51 ± 0.01 vs. 0.48 ± 0.02 vs. 0.48 ± 0.02 g cm^-3^, respectively). A significant interaction between population and WR treatment was found for relative growth rate and net assimilation rate (**Table 3**). The interaction was similar: WR treatment significantly decreased rates in Cayumanque and Nacimiento while Santiago was unaffected (**Figures 7A, C** **)**.

**Figure 8:**
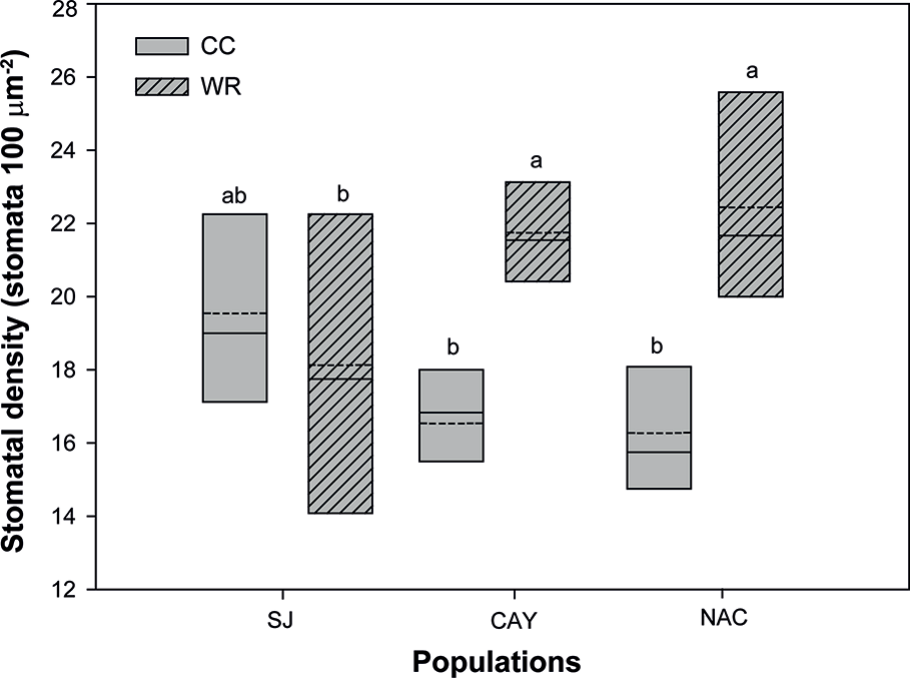
Population and water restriction treatment significantly interacted to affect stomatal density of *Persea lingue* plants. Water restriction (WR) increased stomatal density in plants from Cayumanque (CAY) and Nacimiento (NAC) compared to plants at container capacity (CC), while it had no effect on Santa Juana (SJ). Bottom and top boundaries of the boxes represent the 25^th^ and 75^th^ percentiles, respectively. The solid line in the center of each box represents the mean value and the dashed line represents the median value. Different letters indicate significant differences among populations and the irrigation treatments according to Tukey at p<0.05.

In *P. lingue*, while biomass and biomass partitioning parameters were unaffected by WR treatment, population had a significant effect on all morphological variables except total leaf area (**Table 3**; see **Supplemental Table 1** for means). As with *C. alba*, we again noted high standard deviations around the means (see **Supplemental Table 1**), large p-values for the various interactions of population, treatment, and measurement time (**Table 3**), and no correlation between individual leaf area and stomatal density for any population (Cayumanque r=0.055, p=0.872; Nacimiento r=0.248, p=0.462; Santa Juana r=-0.272, p=0.455). We did, however, observe a significant interaction of population and WR treatment (p=0.0004) on stomatal density. WR significantly increased density in Cayumanque and Nacimiento compared to CC plants but had no effect on Santa Juana (**Figure 8**). Total biomass was significantly higher in Cayumanque than in Nacimiento, followed by Santa Juana (**Figure 6B**), and accordingly, biomass of leaves, stems, and roots followed the same trend as total biomass among populations. Similarly, individual leaf area was significantly higher in Cayumanque (15.19 ± 3.84 cm^2^ leaf^-1^) than in Nacimiento and Santa Juana (11.65 ± 2.54 and 12.46 ± 5.30 cm^2^ leaf^-1^, respectively). For biomass partitioning, Cayumanque presented significantly higher leaf mass ratio and lower root mass ratio compared to Nacimiento and Santa Juana, while stem mass ratio was unaffected by population (**Figure 6F**). Specific leaf area was also influenced by population, Nacimiento displayed a lower value than Cayumanque and Santa Juana (**Figure 6D**). In contrast, Nacimiento displayed a higher wood density than Cayumanque and Santa Juana (0.49 ± 0.01 vs. 0.46 ± 0.01 vs. 0.47 ± 0.01 g cm^-3^, respectively). Relative growth rate was significantly affected by a WR treatment and population interaction (**Table 3**); plants from Cayumanque and Nacimiento were unaffected by this treatment but the rate was significantly decreased in plants from Santa Juana (**Figure 7B**). The WR treatment caused a significant (p=0.012) increase in net assimilation rate compared to CC plants (0.217 ± 0.03 vs. 0.127 ± 0.03 mg cm^-2^ day^-1^, respectively), and plants from Cayumanque and Nacimiento showed significantly (p=0.002) higher net assimilation rate than those from Santa Juana (**Figure 7D**).

## Discussion

In both species, we found significant differences in plant physiology, morphology, and growth among populations before the application of WR treatment. This was especially pronounced in *C. alba* compared to *P. lingue*; we attribute this to the greater geographic distances between *C. alba* populations compared to *P. lingue*.

In *C. alba*, the most xeric population (Santiago) displayed lower A_N_, gs, and E before the WR treatment application, which is in agreement with smaller plants in terms of total biomass, lower relative growth, and net assimilation rates compared to the most mesic population (Cayumanque). Similar differences in size and growth among mesic and xeric populations were observed in species such as *Populus davidiana* (Zhang et al., 2004), *Eucalyptus microtheca* (Li, 2000), and *Pinus ponderosa* (Kerr et al., 2015). Additionally, the most xeric *C. alba* populations displayed higher specific leaf area in contrast to the mesic population. Even though lower specific leaf area is characteristic of tree species with sclerophyllous leaves from xeric sites (Bussotti et al., 2002; Marchín et al., 2008), this trait carries an increased leaf construction cost (Villar et al., 2006; Vico et al., 2015). Thus, cheaper mesophyllous leaves along with the observed lower wood density could constitute a saving strategy for *C. alba* seedlings in more xeric sites, which concurs with observations in other Mediterranean species (Li, 2000; Zhang et al., 2004; Haramaya et al., 2016; Ramírez-Valiente et al., 2010). Such characteristics in *C. alba* plants from xeric sites, are consistent with a resource-conservative strategy and increased drought tolerance (Wright et al., 2010; Reich, 2014).

In relation to the response of *C. alba* to WR, changes in physiology, morphology, and growth parameters were also observed among populations. A decrease in A_N_, gs, and E was observed in all *C. alba* populations under the WR treatment, which has been extensively reported for Mediterranean species (Gulías et al., 2002; Gimeno et al., 2008; Lázaro-Nogal et al., 2013). Thus, the concomitant decrease in gs and E with an increase in stomatal density could be related to a better control of transpiration during drought (Paoletti and Gellini, 1993), as well as a desiccation-avoiding strategy that is coherent with the rapid stomatal closure and maintenance of high relative water content in response to lower water availability observed in *C. alba*. Given that total leaf area was unaffected by the WR treatment, the greater stomatal density we measured on fully expanded leaves at the end of the experiment could be considered an active response to the WR treatment. Although morphological traits were unaffected by the WR treatment, we observed lower individual leaf area (smaller leaves) in plants from the xeric population (Santiago), which is linked to drought resistance and higher WUE (Ackerly et al., 2001). In agreement, we observed a plastic response, seen as the interaction between population *x* irrigation treatment, in iWUE among *C. alba* populations (**Figure 2A**), indicating that iWUE response to the WR treatment is population specific. While an increase in iWUE was observed in plants from xeric populations, which is expected in plants under drought (Gulías et al., 2002; Valladares and Pearcy, 2002; Valladares et al., 2005) and in plants from more xeric climates (Gratani et al., 2003; Figueroa et al., 2010; Kerr et al., 2015; Viger et al., 2016), this was not observed in plants from the most mesic population. Thus, the most xeric population (Santiago) was the least affected by WR with a reduction in A_N_ and gs of 48% and 68%, respectively. While in Nacimiento the increase in iWUE was mainly due to stronger decrease in gs in the WR treatment, with respect to CC plants (88%). In accordance with iWUE results, we also observed a population specific response to lower water availability for relative growth and net assimilation rates, indicating plasticity in growth among *C. alba* populations. Under the WR treatment, neither relative growth nor net assimilation rates for the most xeric population were affected, in contrast to the less xeric (Nacimiento) and mesic (Cayumanque) populations. For the less xeric and mesic populations, a pronounced decrease in A_N_ and gs during WR could have limited carbon assimilation, thus negatively affecting growth parameters in both populations. Also, the photosynthetic machinery of the most xeric population was minimally affected by WR with only a transient decrease in ϕPSII and an increment in ETR, results previously observed in Mediterranean tree species in response to drought (Martínez-Ferri et al., 2000; Valladares et al., 2005; Nguyen et al., 2017), suggesting higher tolerance of the primary processes of photosynthesis to WR in populations from xeric climate. Conversely, plants from the mesic population displayed an activation of photoprotective mechanisms as heat dissipation in WR treatment, observed through an increase in NPQ, revealing an impact of WR on photochemistry in this population.

In regard to morphology, including biomass partitioning, all *C. alba* populations responded equally to the WR treatment; this behavior in morphological features with drought has been previously reported for Mediterranean species (Gimeno et al., 2008; Ramírez-Valiente et al., 2010; Ramírez-Valiente and Cavenders-Bares, 2017). According to Gimeno et al. (2008), leaf morphology and biomass partitioning could be more involved in local adaptation, while physiological changes could be plastic enough to cope with changes in water availability. This agrees with results observed in *C. alba*, where population specific responses were observed on physiological traits, such as iWUE. In general, *C. alba* populations responded to reduced water availability with increased root mass ratio and a decrease in leaf biomass. Greater biomass partitioning towards root and a decrease in organs involved in transpiration is a common response to drought for many plant species (Schall et al., 2012). Especially, an increase in root mass ratio is key in coping with drought because more roots in relation to the shoot improve the capacity to explore deeper soil layers for water (Markesteijn and Poorter, 2009; Matías and Jump, 2014), and enhances establishment success during the first drought season. An increase in wood density under WR, which has been previously observed in tree species (Stiller, 2009; Corcuera et al., 2011), could reduce the risk of cavitation under negative pressure imposed by lower water availability (Hacke et al., 2001; Aranda et al., 2015).

Finally, improved iWUE and growth response in plants from the most xeric *C. alba* population could induce an advantage in competition and survival for seedlings as they experience their first summer drought that is characteristic of Mediterranean environments (Gimeno et al., 2008), and could increase resilience and acclimation capacity in future scenarios of increased aridity. In *C. alba*, iWUE and growth rates could be used as possible parameters to differentiate population responses to drought events, while morphological traits could be related to local adaptation.

Similar to *C. alba*, *P. lingue* plants displayed physiological differences among populations before the application of WR. Differences in physiological traits were, however, not related to environmental characteristics of the populations of origin. Thus, higher A_N_ and gs was observed in Cayumanque plants, which is consistent with bigger plants with higher total biomass. The *P. lingue* plants from the most mesic population (Santa Juana) presented more mesophyllous leaves with higher specific leaf area, which is a common trait in plants from sites with increased rainfall (Fonseca et al., 2000; Bussotti et al., 2002; Marchín et al., 2008). Even though higher photosynthetic rates are expected in such mesophyllous leaves (Wright et al., 2004), Flexas et al. (2014) indicated that a negative correlation between specific leaf area and photosynthesis could be related to changes in Rubisco carboxylation velocity. Thus, diffusive or biochemical limitations to Rubisco carboxylation velocity could also explain differences among population photosynthesis rates, which is in agreement with lower A_N_ in Santa Juana plants despite higher Ci compared to the other populations (Gulías et al., 2002; Peña-Rojas et al., 2004). In contrast seedlings from the most xeric population (Nacimiento) presented a lower specific leaf area compared to the more mesic population, which was linked to less leaf biomass and a lower leaf area ratio. These are traits commonly observed in plants from drier climates as a strategy associated to limit water loss through a reduction in transpirational tissue (Poorter and Markesteijn, 2008; Peguero-Pina et al., 2014).

In contrast to *C. alba*, we observed that WR minimally influenced physiological and morphological parameters in *P. lingue*. Only A_N_ and stomatal density displayed a plastic response among *P. lingue* populations during the WR treatment, while no change was observed on morphological traits. However, as mentioned before, *P. lingue* populations were geographically restricted to the northernmost distribution of the species, so different responses could be found if more broadly distributed populations were included in further analysis. It is worth noticing that *P. lingue* plants reached higher VSWC values in the WR treatment compared to *C. alba* (53.7 ± 1.2% vs. 43.0 ± 0.8%), which could have induced a minor physiological adjustment in response to WR treatment. Also, WR treated *C. alba* plants had higher gs and E than *P. lingue* plants (0.019 ± 0.003 mol m^-2^s^-1^ and 0.227 ± 0.04 mmol H_2_O m^-2^s^-1^ vs. 0.01 ± 0.005 mol m^-2^s^-1^ and 0.164 ± 0.12 mmol H_2_O m^-2^s^-1^, respectively); such higher transpiration rates in *C. alba* may have led to lower VSWC than that observed for *P. lingue*. The most xeric populations of *P. lingue* presented an increase in stomatal density, which could contribute to the control of transpiration and iWUE (Nilson and Assmann, 2007; Roussel et al., 2009). This result has, however, no apparent relation with gs, E, or iWUE in this particular species, indicating a poor control of water loss during WR despite a decrease in plant water status (Ψ_min_) (Sánchez-Vilasand Retuerto, 2007; McDowell et al., 2008; Viger et al., 2016). Nonetheless, we believe our observed changes in stomatal density in response to WR could be considered an active population-specific response, independent of individual leaf area. The most mesic population was negatively affected by WR application, inducing a decrease in A_N_ and relative growth rate, but with no adjustment in the control of water loss observed through changes on gs, E, or iWUE.

Similar to *C. alba*, fluorescence parameters were slightly affected by WR in *P. lingue*; F_v_/F_m_ was only affected by population (data not shown) but mean values were above 0.8 in all populations. Water restriction affected fluorescence parameters mainly in Cayumanque, a slight decrease in ETR, qP, and ΦPSII was observed in WR plants, indicative of lower capacity of excess energy dissipation through photochemistry, whereas an increase in NPQ indicates activation of photoprotective mechanisms inducing higher energy dissipation as heat. These results regarding the *P. lingue* response to the WR treatment indicate that this species has a drought-tolerant strategy to cope with water scarcity in most xeric populations, which allows plants to maintain physiological function and growth despite a decrease in Ψ_min_ (Martínez-Ferri et al., 2000; Gulías et al., 2002; Meinzer et al., 2014).

Our study revealed that the drought response of *C. alba* and *P. lingue* diverge among populations, in relation to physiological and morphological adjustments. For both species, however, physiological parameters were more responsive to WR treatment than morphological traits, including biomass partitioning. Thus, it seems that physiological adjustments sufficiently plastic to cope with environmental changes imposed in this experiment (Gimeno et al., 2008). Our results indicate that differences between populations in morphological traits are involved in local adaptation processes and can be driven by differences in climate of the original environment (Bussotti et al., 2002; Ramírez-Valiente et al., 2010; Kaluthota et al., 2015; Ramírez-Valiente and Cavender-Bares, 2017). These results concur with Poorter and Nagel (2000) who demonstrated that biomass allocation changes are smaller during water limitation compared to scarcity of other resources such as light and nutrients.

## Conclusions

Our results reveal inter-population differences are evident at the physiological and morphological levels for *C. alba* and *P. lingue*. Differences in morpho-physiological traits in *C. alba* were related to the climatic characteristics of the origin of the populations, indicating that plants from xeric climate present a resource-conservative strategy. In contrast, morpho-physiological differences among *P. lingue* populations were subtle and not clearly linked to climate conditions, which we attribute to the restricted distribution of the populations we selected.

For both species, physiological and growth adjustments were more responsive to the WR treatment than were morphological traits. In response to decreased water availability, the most xeric *C. alba* population performed better under stressful conditions in terms of growth and iWUE. Thus, relative and net assimilation rates with iWUE were effectively capable of differentiate population responses upon WR treatment. Despite that, differences in WR response among *P. lingue* populations were subtle, most xeric populations also performed better under stress, which we observed mainly in relation to relative growth rate response. Growth is an important trait to consider during water stress, as higher growth rates in early life will allow increased survival and establishment success under Mediterranean climates with higher changes of drought events. This research provided evidence regarding different population performances during WR in *C. alba* and *P. lingue* that could assist in the selection of populations with better chances of survival in regions with projected increases in climatic stress. Future research should, however, assess additional populations, aiming to include the complete range of distribution of each species to understand the potential suitability of different populations to be considered for restoration efforts.

While seed transfer should always be considered in restoration efforts, it may be more critical in the face of climate change. Unfortunately, we often lack information on site-adapted ecotypes according to target environment and future climatic patterns, such as an increase in drought events. Our results begin to bridge that information gap, as they suggest that the Santiago population could be a candidate, site-adapted seed source for restoration on more southerly sites. This approach should only be implemented, however, after considering variation in genetic diversity and differentiation (e.g. Hasbún et al., 2016).

## Data Availability Statement

The datasets generated for this study are available on request to the corresponding author.

## Author Contributions

Conceived and designed the experiment: CA-M, MA, MG. Collected plant material from populations: CA-M, MA, MG, EC. Performed experiments: CA-M. Performed data analysis: MA. Wrote the manuscript: CA-M, KD. Revised manuscript language: KD.

## Funding

Chilean Environmental Ministry and CONICYT research project I786010006.

## Conflict of Interest

The authors declare that the research was conducted in the absence of any commercial or financial relationships that could be construed as a potential conflict of interest.
