# Supplementary material for: Intraspecific Variation in Drought Response of Three Populations of Cryptocarya alba and Persea lingue, Two Native Species From Mediterranean Central Chile
Source: Front Plant Sci. 2020 Jul 16;11:1042. doi: 10.3389/fpls.2020.01042 (PMC7378861; doi:10.3389/fpls.2020.01042)
Supplement: Supplementary file 1 [file DataSheet_1.docx]

**Supplementary information**

**
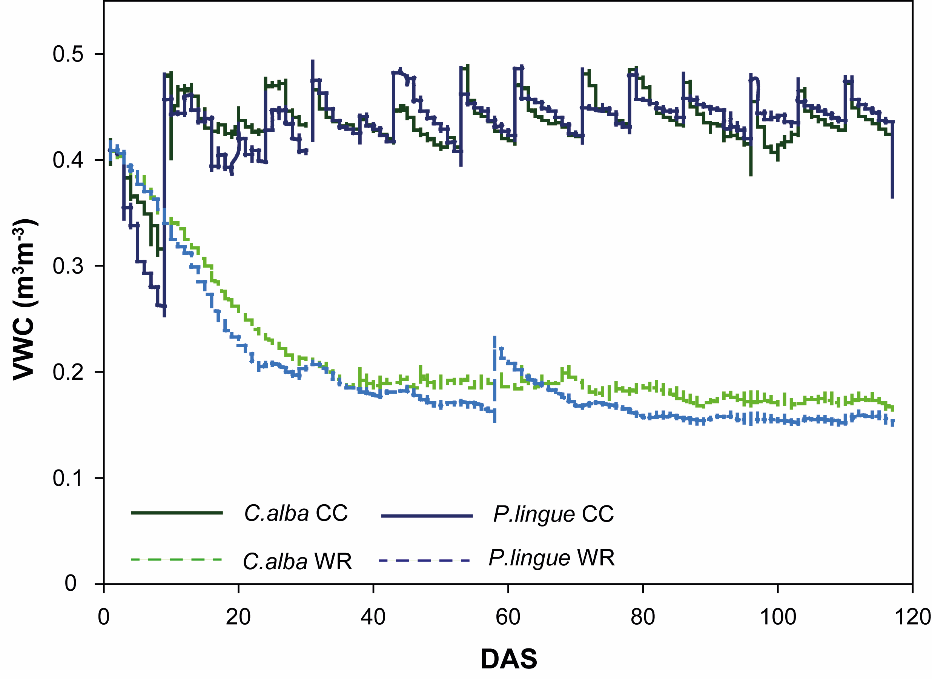
**

**Supplementary figure 1.** Evolution of volumetric soil water content (m^3^ m^-3^) during water restriction experiment in *Cryptocarya alba* at container capacity (CC) (dark green) and under water restriction (WR) (light green) and *Persea lingue* at CC (dark blue) and WR (light blue). VWC: Volumetric water content, DAS: days after onset of stress treatment.

**Supplementary table 1.** Summary of morphological variables (mean ± standard deviation) for *Cryptocarya alba* and *Persea lingue* plants during water restriction experiment. M.T: measurement time; LDM: leaf dry mass; SDM: shoot dry mass; RDM: root dry mass; LA: total leaf area; ILA: individual leaf area; TM: total plant biomass; WD: wood biomass; CC: container capacity treatment and WR: water restriction treatment

| Population |  | Treatment | LDM (g) | SDM (g) | RDM (g) | LA (cm^2^) | ILA (cm^2^leaf^-1^) | TM (g) | WD (g cm^-3^) |
| --- | --- | --- | --- | --- | --- | --- | --- | --- | --- |
| *Cryptocarya alba* | | | | | | | | | |
| Cayumanque | Pre-WR | CC | 4.81±0.47 | 2.16±0.26 | 4.58±0.41 | 488.6±54.26 | 5.70±0.78 | 11.55±1.10 | 0.45±0.01 |
|  | Post-WR | CC | 4.33±0.18 | 2.90±0.30 | 6.30±0.78 | 407.1±14.26 | 4.91±0.37 | 13.53±0.87 | 0.51±0.01 |
|  |  | WR | 3.21±0.27 | 1.76±0.16 | 4.81±0.40 | 285.0±23.01 | 5.29±0.45 | 9.79±0.75 | 0.62±0.01 |
| Nacimiento | Pre-WR | CC | 4.48±0.35 | 2.61±0.26 | 4.41±0.57 | 476.3±21.42 | 6.97±0.43 | 11.50±1.08 | 0.43±0.01 |
|  | Post-WR | CC | 7.33±0.45 | 4.76±0.45 | 8.21±0.67 | 809.6±115.33 | 8.87±0.90 | 20.29±1.37 | 0.5±0.02 |
|  |  | WR | 5.11±0.55 | 3.28±0.34 | 7.24±0.61 | 502.8±60.04 | 6.43±0.57 | 15.62±1.34 | 0.59±0.01 |
| Santiago | Pre-WR | CC | 3.42±0.34 | 1.94±0.19 | 3.19±0.35 | 398.9±46.35 | 3.90±0.44 | 8.56±0.85 | 0.43±0.02 |
|  | Post-WR | CC | 3.50±0.15 | 2.37±0.22 | 4.08±0.30 | 355.3±25.51 | 3.79±0.19 | 9.95±0.45 | 0.46±0.02 |
|  |  | WR | 3.57±0.28 | 2.75±0.21 | 5.29±0.32 | 338.0±24.45 | 3.47±0.26 | 11.61±0.78 | 0.6±0.01 |
| *Persea lingue* | | | | | | | | |  |
| Santa Juana | Pre-WR | CC | 3.43±0.38 | 2.53±0.27 | 5.16±0.39 | 333.73±29.93 | 10.52±1.05 | 11.11±0.87 | 0.49±0.01 |
|  | Post-WR | CC | 3.57±0.60 | 2.11±0.30 | 6.05±0.98 | 343.50±44.93 | 13.96±3.78 | 11.73±1.79 | 0.48±0.01 |
|  |  | WR | 4.04±0.96 | 2.69±0.41 | 8.47±1.20 | 387.51±111.18 | 14.85±1.40 | 15.20±2.52 | 0.48±0.01 |
| Cayumanque | Pre-WR | CC | 5.68±1.45 | 3.68±0.91 | 5.58±0.97 | 543.36±129.83 | 14.61±1.33 | 14.93±3.29 | 0.45±0.01 |
|  | Post-WR | CC | 6.32±0.82 | 3.99±0.32 | 9.13±1.18 | 555.65±73.93 | 16.56±2.30 | 19.45±1.99 | 0.48±0.01 |
|  |  | WR | 7.26±1.07 | 5.43±0.81 | 12.01±1.6 | 575.66±63.53 | 14.98±1.40 | 24.70±3.38 | 0.48±0.01 |
| Nacimiento | Pre-WR | CC | 4.45±0.51 | 2.97±0.34 | 5.73±0.84 | 329.63±38.06 | 12.13±0.70 | 13.15±1.58 | 0.48±0.01 |
|  | Post-WR | CC | 4.81±0.75 | 3.88±0.46 | 9.18±0.99 | 396.13±64.50 | 10.21±1.47 | 17.88±2.01 | 0.51±0.01 |
|  |  | WR | 4.52±0.60 | 3.59±0.46 | 10.18±1.3 | 365.53±39.15 | 12.12±1.12 | 18.29±2.31 | 0.49±0.01 |
